# Cimetidine in colorectal cancer – are the effects immunological or adhesion-mediated?

**DOI:** 10.1038/sj.bjc.6600097

**Published:** 2002-01-21

**Authors:** D Eaton, R E Hawkins

**Affiliations:** CRC Department of Medical Oncology, Christie Hospital NHS Trust, Wilmslow Rd, Manchester, UK

## Abstract

*British Journal of Cancer* (2002) **86**, 159–160. DOI: 10.1038/sj/bjc/6600097 www.bjcancer.com

© 2002 The Cancer Research Campaign

## 

In this issue Matsumoto *et al* (2002) report the 10 year survival rates of a randomized placebo-controlled trial in which colorectal cancer patients received cimetidine and oral 5-fluorouracil (5-FU) or 5-FU alone as an adjuvant therapy following curative resection. In this small study a highly significant improvement in survival is demonstrated most notably in patients with node-positive (Dukes C) tumours (10 year survival – 85%). A further retrospective analysis suggests that this appeared to be due to a remarkable effect of cimetidine on patients with tumours expressing the sialyl Lewis adhesion epitopes X and A (sL^x^ and sL^A^).

The histamine type 2 (H_2_) receptor antagonist cimetidine was first proposed as an anti-cancer agent in 1979 (Armitage and Sidner, 1979). This followed the spontaneous remission of two patients with metastatic carcinoma after coincidental use of the drug. Since then there have been several clinical trials assessing cimetidine in a range of malignancies with varied and inconclusive results.

In 1988 it was reported that post-operative treatment with cimetidine improved survival in gastric cancer patients of all stages (increasing median survival from 316 to 459 days) (Tonnesen *et al*, 1988). However, in a more recent and larger study organized by the British Stomach Cancer Group, cimetidine had no effect on survival when compared to placebo (Langman *et al*, 1999). Trials combining cimetidine with immunotherapy approaches in renal cell carcinoma and melanoma have also failed to demonstrate any benefit to the addition of cimetidine (Creagan *et al*, 1985; Sagaster *et al*, 1995).

Results have been more promising using cimetidine in colorectal cancer. In addition to the study in this issue (which was first reported in 1995; Matsumoto, 1995), there are now three other studies that have shown non-significant trends to improved survival. In 1994 Adams and Morris reported that a 7 day perioperative course of cimetidine improved 3-year survival from 59 to 93% (*P*=0.17) in 34 colorectal cancer patients (Adams and Morris, 1994). In a more recent study of 125 patients by the same group this regime showed a similar trend that achieved significance in patients with tumours negative for microsatellite instability (Kelly *et al*, 1999). In a randomized study of 192 patients, Svendsen *et al* (1995) also showed a trend to improved survival in Dukes C patients. In addition to these studies of adjuvant cimetidine, a small trial compared 5-FU-based chemotherapy alone or in combination with cimetidine in patients with advance disease (Links *et al*, 1995). Although no difference in overall response was seen there was a significantly increased rate of CEA response (>50% reduction compared with baseline) in the cimetidine group.

The exact mechanism by which cimetidine may exert an anti-cancer effect remains uncertain. High concentrations of histamine are known to occur in colorectal cancer tissues (Garcia-Caballero *et al*, 1993). Histamine stimulates the *in vivo* growth of experimental colorectal tumours and cimetidine can inhibit this effect (Adams *et al*, 1994a). However, other, far more potent H_2_ receptor antagonists such as ranitidine do not show the same effect either *in vitro* (Lawson *et al*, 1996), or in large clinical trials (Nielsen *et al*, 1998). This suggests that either the H_2_ receptor on tumour cells is structurally distinct from those found on parietal cells, or that the mechanism of action my be independent of classical H_2_ receptor antagonism.

Cimetidine has also been shown to have a number of immunomodulatory effect. Histamine negatively regulates T helper (T_H_1 and T_H_2) cell responses through the H_2_ receptor and H_2_ receptor knockout mice show upregulation of T_H_1 and T_H_2 cytokines (Jutel *et al*, 2001). Increased release of histamine has been proposed as the cause of the immunosuppression seen at the time of colonic resection and several studies have shown that perioperative H_2_ receptor antagonists can prevent this effect, thereby improving immune surveillance at the time of surgery (Adams *et al*, 1994b). The presence of tumour infiltrating lymphocytes (TIL) within rectal tumour tissues is considered to be an independent pathological marker of good prognosis (Harrison *et al*, 1994). Early clinical studies suggested that perioperative treatment with cimetidine resulted in an increased peritumoural lymphocyte infiltration (Adams and Morris, 1994, 1997). A more recent trial however, has failed to confirm this with the subgroup of patients appearing to benefit most from cimetidine being the aggressive tumours that lacked TILs (Kelly *et al*, 1999).

Recently Kobayashi *et al* (2000) have proposed a novel mechanism of action. They showed that cimetidine could block the adhesion of a colorectal tumour cell line to endothelial cells *in vitro* and could suppress the formation of hepatic metastases in a nude mouse model, via the down regulation of cell surface expression of the adhesion molecule E-selectin on endothelial cells.

E-selectin (or ELAM-1) is a member of the selectin family of adhesion molecules. Expression of E-selectin is induced on the cell surface of activated endothelial cells by key mediators of inflammation such as Il-1β and TNF-α (Bevilacqua *et al*, 1987) and has also been reported on the surface of tumour endothelial cells (Ye *et al*, 1995). Cell adhesion to E-selectin is mediated through the carbohydrate ligands sL^X^ and sL^A^ which are expressed predominantly on neutrophils (Phillips *et al*, 1990). In this way neutrophils can be recruited to sites of inflammation. However, sL^X^ and sL^A^ are known to also be expressed on a number of different tumour cell types and for many years this interaction has been implicated in the pathogenesis of cancer metastasis (Brodt *et al*, 1997; Dennis *et al*, 1982; Kitayama *et al*, 2000). SL^X^ expression is increased in human hepatic colorectal metastases compared to the primary tumour (Hoff *et al*, 1989) and expression has been shown to correlate with poor survival of colorectal cancer patients (for example, in stage III patients 5 year survival was 42% in the sL^X^ positive group *vs* 81% in patients with tumours negative for sL^X^ in one study) (Nakamori *et al*, 1993). Expression of E-selectin on endothelial cells has also recently been shown to be essential in models of transendothelial migration of colon carcinoma cells (Laferriere *et al*, 2001).

Matsumoto *et al* (2002) have also retrospectively analyzed the patients in their previous study of cimetidine in colorectal cancer according to sL^A^ and sL^X^ expression. This was a small study the primary endpoints of which were originally to improve appetite and reduce oesophagitis, not prolong survival. In addition, it has not been analyzed on an intention to treat basis (10% of patients were excluded from analysis). The results, however, are striking and do appear to justify further investigation of the effect of cimetidine on E-selectin expression and the consequences of this interaction on sL^A^ and sL^X^ expressing colonic carcinoma.

In summary, there appears to be beneficial effects of cimetidine on colorectal cancer. There is some evidence for immunological mechanisms and for effects on E-selectin mediated adhesion. Further large-scale clinical trials appear warranted.
